# Judgement bias may be explained by shifts in stimulus response curves

**DOI:** 10.1098/rsos.221322

**Published:** 2023-04-05

**Authors:** Caroline Strang, Felicity Muth

**Affiliations:** ^1^ Department of Integrative Biology, University of Texas at Austin, Austin, TX 78712, USA; ^2^ School of Behavioural and Social Sciences, Brescia University College, London, Ontario, Canada N6G 1H2

**Keywords:** cognitive bias, optimism, peak shift, *Bombus impatiens*, bumblebee, bee

## Abstract

Judgement bias, or ‘optimism’ and ‘pessimism’, has been demonstrated across many taxa, yet the cognitive mechanisms underlying this behaviour remain unclear. In an optimism paradigm, animals are trained to an association, and, if given a positive experience, behave more favourably towards ‘ambiguous’ stimuli. We tested whether this effect could be explained by changes to stimulus response gradients by giving bees a task where their response was tested across a wider gradient of stimuli than typically tested. In line with previous work, we found that bees given a positive experience demonstrated judgement bias, being more likely to visit ambiguous stimuli. However, bees were also less likely to visit a stimulus on the other side of the rewarded stimulus (S+), and as such had a shifted stimulus response curve, showing a diminished peak shift response. In two follow-up experiments we tested the hypothesis that our manipulation altered bees’ stimulus response curves via changes to the peak shift response by reducing peak shift in controls. We found that, in support of our hypothesis, elimination of peak shift also eliminated differences between treatments. Our results point towards a cognitive explanation of ‘optimistic’ behaviour in non-human animals and offer a new paradigm for considering emotion-like states.

## 1. Introduction

Although difficult to define [1], emotions have been described as ‘suites of cognitive, motivational and physiological changes that are triggered by appraisal of specific classes of environmental situations’ [2]. One way that emotional states have been studied is through judgement bias tasks [3,4] where an individual is trained via differential conditioning to a rewarded stimulus (S+) and unrewarded or punishing stimulus (S−) before undergoing a particular experience (either expected to induce a negative or positive affective state). The subject is subsequently tested on their response towards the S+, S− and ‘ambiguous’ stimuli that lie between the two trained stimuli [5]. A diminished or enhanced response to the ambiguous stimulus is described as ‘pessimistic’, or ‘optimistic’ bias, respectively [4]. The terms ‘pessimistic’ and ‘optimistic’ are operationalized as behavioural responses to ambiguous stimuli and extrapolated to represent emotion-like states [3]. Judgement bias tasks have been used most widely in animal welfare studies where they often serve as indicators of animals’ affective states in response to an experience such as a stressful housing condition or environmental enrichment [6,7].

The first demonstration of judgement bias in a non-human animal was in rats [4], and since then judgement biases have been shown in a number of vertebrates, including European starlings *Sturnus vulgaris* [8,9], common ravens *Corvus corax* [10], rhesus macaques *Macaca mulatta* [11] and collared peccaries *Pecari tajacu* [12]). Indeed, a meta-analysis in 2020 identified 71 studies in 22 non-human species [6], finding that while most experiments used mammals, judgement biases have been demonstrated across broad taxa. The honeybee was the first invertebrate shown to demonstrate judgement bias, showing a ‘pessimistic’ response to a stressful event (being shaken) [5], see also [13]. More recently, bees were also shown to express positive biases: bumblebees that received an ‘unexpected reward’ took less time to visit an ambiguous visual stimulus, while responses to the S+ and S− remained the same [14].

While the behaviour apparent in judgement bias tasks has been well-documented, and is robust across species and experimental treatments [6], the cognitive mechanisms underlying this behaviour have not been investigated. On the one hand, it has been suggested that judgement bias effects cannot be explained by general sensory or motivational changes because responses to trained cues (S+ and/or S−) often remain the same [3,5]. Others have argued that judgement biases may be explained by what is already known about learning and/or motivational mechanisms [15,16]. Indeed, classic work in experimental psychology tells us that variables such as motivation can alter the shape of stimulus response curves without changing the peak [17,18]. As such, shifts in stimulus response generalization curves may explain how animals may change their response to an ambiguous stimulus, while responses to the S+ and S− remain the same (example in electronic supplementary material, figure S1). Similar ideas have also been proposed in [19].

**Figure 1.**
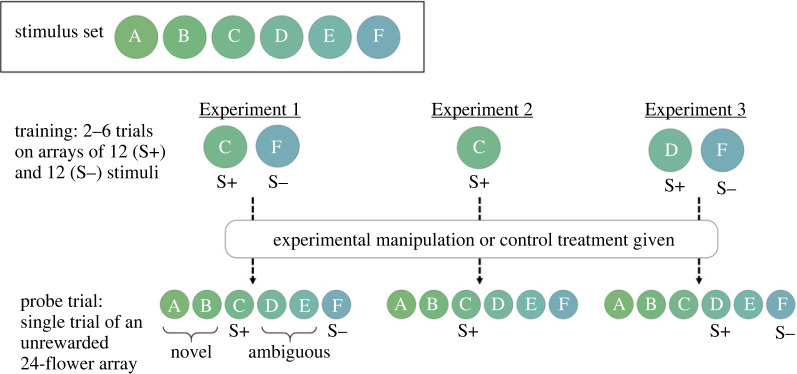
Diagram of the experimental design and colour stimuli used in the current experiments. Six colour stimuli (indicated by letter; (*a–f*) were used across all experiments; each stimulus was roughly equidistant from the previous in bee colour space (electronic supplementary material, table S1). In each experiment, bees were first trained to learn a colour association across two to six training trials; S+ indicates rewarding flower type; S− indicates unrewarding flower type. Individuals were then either given a reward (5 µl of 50% sucrose) in the experimental treatment or no reward in the control treatment. All bees were then tested for their preferences on an unrewarding probe array where they were presented with 24 flowers, with four of each colour.

Here, we addressed how positive emotion-like states could be explained at a psychophysical level, using bumblebees as a model [20–22]. When learning a discrimination, animals typically form a response curve around the S+, generalizing their response to similar stimuli [23], including ambiguous stimuli, and stimuli farther from the S+; hereafter ‘novel’ stimuli (electronic supplementary material, figure S1). We refer to these stimuli as ‘ambiguous’ and ‘novel’ in line with existing terminology, while acknowledging that ‘ambiguous stimuli’ are also novel to trained bees, and that the ‘novel’ stimuli may be ambiguous to bees in that they have not been reinforced or unreinforced via training. Based on previous work showing that motivational changes can alter the shape of stimulus response curves [17,18], we hypothesized that ‘positive’ experiences would broaden the stimulus response curve, thus increasing responsiveness towards the ambiguous stimuli, but also to novel stimuli (electronic supplementary material, figure S1*a*). Conversely, negative experiences (not tested here) may narrow the range of stimuli that an animal is willing to accept (electronic supplementary material, figure S1*b*). Importantly, such changes would lead to similar responses to the S+ and S−, a key aspect of judgement bias. We initially tested this hypothesis in Experiment 1 using a modified judgement bias task that expanded the range of stimuli typically used. We first trained bees to a S+/S− discrimination, then assigned them to control or experimental groups, the latter of which received a sucrose reward (as in the previous study on positive emotion-like states in bumblebees [14]), before testing them on a range of stimuli in a single probe trial that included the S+, S−, ambiguous and novel stimuli (figure 1). Past work on judgement biases has most often used a ‘go/ no-go’ design, where the animal is presented with a single stimulus in a probe trial and either responds or withholds their response, while other studies have used an ‘active choice’ design where the animal is presented with a stimulus in a probe trial and must select between two trained responses which correspond to the S+ and S− [6]. In both cases, the number of sequential presentations in the probe trial varies greatly among studies, as well as whether stimuli are reinforced or not [6]. In our study, we used a design adopted from work on generalization gradients [24,25], but new in the context of measuring judgement bias, where we presented bees with an array of multiple stimuli simultaneously in order to rapidly assess preference across a stimulus gradient.

In Experiment 1, we found that a ‘positive’ experience of a reward given after learning appeared to alter bees’ stimulus response curves via changes to the peak shift response (see *Experiment 1 results and discussion*). In two follow-up experiments, we then tested the hypothesis that an increase in preference for ambiguous stimuli following this ‘positive’ experience is explained by a reduction or elimination of the peak shift response. In Experiment 2, we trained individuals via absolute conditioning, thus eliminating the inhibitory curve and peak shift. In Experiment 3, we trained individuals to stimuli closer to each other, which may have increased or decreased peak shift (see below). If our hypothesis held, then we predicted that if we eliminated peak shift, we would no longer see differences between treatments. Conversely, if we had increased peak shift, we would expect to see a larger difference between treatments.

## 2. Material and methods

### 2.1. General methods

#### 2.1.1. Subjects, housing and maintenance

We used worker bumblebees (*Bombus impatiens*) from commercially produced colonies (Koppert, USA). In each experiment, we used 32 (*n* = 16 control; *n* = 16 experimental) foragers, taken equally from two colonies (i.e. 96 bees total). Colonies were tested sequentially, attached to ‘foraging arenas’ (*l* × *w* × *h* = 122 × 61 × 61 cm) via an ‘entrance tube’ (length = 360 cm, diameter = 1.5 cm) where individual bees were presented with vertical arrays of 24 artificial flowers. The entrance tube consisted of a ‘walkway’: a 3 cm diameter clear plastic tube, attached to a 3 cm diameter mesh tube and a ‘holding area’: a 1.5 cm diameter Perspex tube (electronic supplementary material, figure S2). The holding area contained a small hole, through which we fed bees in the experimental procedure. Both the entrance tube and holding area contained metal ‘gates’ that could be raised and lowered to allow individual bees through the tube and to hold individuals in the holding area. The foraging arena was illuminated by fluorescent room lighting and a 40 W LED white light (Commercial Electric, USA) placed directly above the foraging arena. All lights were on a 12/12 h light/dark schedule.

On training days when insufficient sucrose was collected, we pipetted 30% sucrose directly into colony honeypots. We supplied colonies with one tablespoon (approx. 3 g) of pollen directly into the colony every 2 days or as needed (based on visual assessment of colony stores).

#### 2.1.2. Training and testing stimuli

The shaping, training, and testing arrays consisted of corrugated plastic panels with 24 artificial flower locations creating 10 × 40 cm arrays. The panels were painted a dark grey colour (no. 2753, BEHR ULTRA, CA, USA). Training and testing arrays consisted of artificial flowers with ‘corollas’ (circular laminated card sized 4 cm in diameter). We used six coloured stimuli, referred to as A–F, over the course of three experiments. These stimuli were designed to be as close to equidistant as possible in bee colour space (electronic supplementary material, figure S3 and table S1). In hue, saturation and luminance (HSL) the hue of the stimuli ranged from 80° (green) to 135° (blue) in 10° increments (i.e. as in [25] and similar to [24]), with the exception of the interval between the 120° and 135° stimuli which was larger to make the stimuli closer to equidistant in bee colour space, with saturation 75% and luminance 150%. Stimuli were printed on an inkjet printer (Epson stylus C88+) on Cotton Fine Art Archival OBA free paper (Pacific Inkjet, USA). We used a Flame UV–VIS spectrometer (Ocean Insight, FL, USA) to measure reflectance of each stimulus and irradiance in the foraging arena. The reflectance measurements were then analysed and mapped into bee colour space [26] taking into account the photoreceptor spectral sensitivities of *B. impatiens* [27], using AVICOL, a program for analysing spectrometric data [28].

In order to limit biasing bumblebees to particular locations, we used six different training arrays, where flower colour location was pseudorandomized across arrays such that the S+ and S− were represented equally in the top and bottom half of the array. We also pseudorandomized the order that we presented arrays to bees across trials, with all orders presented an equal number of times for control and experimental bees.

For the probe trial array, the stimuli were arranged pseudorandomly such that stimuli of the same colour were never directly beside each other and so that there was equal representation of the S+ and S− stimuli in the top and bottom half of the array, to control for possible location preferences. In Experiment 1, we used four different arrays in the probe phase, which were used an equal number of times across the control and experimental conditions. After one probe array was damaged during the first experiment and we were unable to replace stimuli, we used only three probe arrays in Experiments 2 and 3. In these experiments, the probe arrays were represented roughly equally across treatments and accounted for in statistical analyses.

#### 2.1.3. Pre-training and shaping

We first ‘pre-trained’ bees to forage in the arena and return to their colony between foraging bouts. To do this, colonies were each given access to a feeder (a 250 ml plastic tub containing a white wick) in the foraging arena. The feeder was initially placed at the entrance to the foraging arena; once bees were regularly visiting the feeder we gradually moved it to the back of the arena where the shaping, training and testing arrays would be placed. During pre-training, we restricted colonies’ access to the foraging arena overnight and removed feeders.

In a ‘shaping’ phase, we trained bees in a step-wise fashion to visit our experimental flowers. To do this, we first placed the ‘shaping array’ at the back of the foraging arena and gave foragers free access to it. This array consisted of 24 equally spaced (2.5 cm) clear Eppendorf tubes, containing cotton wicks soaked in 30% (w/w) sucrose. The wicks were replenished as needed to create a constant sucrose supply in all artificial flowers. Once bees foraged on the array, we gradually pulled the wicks back into the flowers until bees had to fully enter the Eppendorf tubes to access the wicks. This part of shaping typically took 1–2 days and was repeated as needed throughout training and testing. Once bees consistently visited the shaping array, we replaced the sucrose-soaked wicks with 40 µl of sucrose, which required bees to fully enter the artificial flowers to access rewards. We replenished flowers throughout the shaping procedure by pipetting sucrose into the Eppendorf tubes via small holes. This latter part of the shaping phase was repeated each day prior to training and testing. To identify individual bees for use in experiments, we paint-marked foragers with a unique colour combination on their thorax with Posca paint pens (Uni Mitsubishi Pencil, USA) during shaping.

#### 2.1.4. Training

In all experiments, paint-marked, shaped bees were alternately assigned to Experimental and Control treatments. During the training phase, all bees were trained to a S+/S− discrimination (Experiments 1 and 3) or S+ conditioning (Experiment 2). Rewarded flowers always contained 10 µl of 30% (w/w) sucrose and unrewarded flowers contained 10 µl of water. After training, bees then underwent the experimental treatment before being tested on a set of six unrewarded stimuli in a probe trial (figure 1). Training phases for each experiment are described in detail below.

#### 2.1.5. Experimental treatment

After training we waited for the bee to leave its colony again to forage, at which point we contained it in a holding area for 2 min, during which time bees in the experimental treatment were fed 5 µl of 50% (w/w) sucrose (as in [14]); control bees were held but not fed. After 2 min, bees were given access to the foraging arena for the probe trial.

#### 2.1.6. Probe trial

During the probe trial, bees were given access to an unrewarded 24-flower array (each flower contained 10 µl of water) (figure 1) for 10 min; after this they were euthanized via freezing.

#### 2.1.7. Behavioural coding and inclusion criteria

We filmed all trials and coded bees’ choice behaviour in probe trials using Solomon Coder [29]. Since flowers were not replenished during learning trials, bees occasionally made visits to previously emptied flowers; these visits were excluded from the learning criterion calculation because it is not clear if bees would perceive such a visit as a CS+ or CS−. In the probe phase, we defined a choice as the bee landing on a flower and inserting its head or body into the flower. We decided to use the first 20 choices bees made as the response measure in the probe phase to allow for sufficient visits to the six stimuli and in line with previous bumblebee cognition experiments [30,31]. Within these 20 choices, bees could visit the same stimulus more than once and it would be counted as a separate choice; we did this to allow a bee to have up to a 100% preference for a given stimulus; otherwise preferences would have been limited to 16.67% (1/6) per stimulus. This decision was informed by observations from similar behavioural experiments that bees will frequently re-visit the same stimulus multiple times in probe phases (F Muth 2014–2022, personal observation). However, we removed bees that made more than 50% of their choices to a single location, since this may indicate a strong location rather than stimulus preference (*n* = 1, see Experiment 1 methods). The majority of bees (81/96) made at least 20 choices in the probe phase, yet we did not exclude bees that made fewer than this; all bees included in the final dataset made at least 12 choices. We did not include all of bees’ choices within the 10-min recorded trial because after 20 unrewarded visits bees may be more likely to shift to random sampling after not receiving rewards.

Previous work on positive judgement bias in bees found that bees given a reward did not differ from controls in their latency to make a choice [14]. We confirmed that this was also the case in the current study by extracting the time it took for bees to make their first choice to a stimulus from recorded videos. We found this measure did not vary between treatments for any experiment; statistical methods and results are in the electronic supplementary material.

#### 2.1.8. Data analysis

All statistical analyses were conducted in R v. 4.1.2 [32]. In all experiments, to determine if treatments differed in their stimuli preferences in the probe phase, we asked if bees in the experimental treatment differed in their choices to stimuli compared with control bees. We did this using linear mixed models (LMMs) (package: nlme; function: lme) [33]. In all analyses, the response variable was the number of visits to a given stimulus (in the first 20 visits), and the explanatory variables included were: ‘Condition’ (treatment or control), ‘Stimulus’ (A, B, C, D, E or F), 'Probe array' (1–4 in Experiment 1 and 1–3 in Experiments 2 and 3) and ‘Bee’ nested within ‘Colony’ as random factors. Bee and Colony were included as random factors in all models because visits to different stimuli were non-independent, i.e. a given bee would visit multiple different stimuli. We initially ran a full model including the three-way interaction between Condition, Stimulus and Probe array, but then removed non-significant interaction terms (*p >* 0.05) in a step-wise fashion while always keeping main effects in the model (per [34]). We used the anova() function to generate *F-*values and *p*-values for model terms. We then used the package emmeans [35] to carry out Tukey *post hoc* comparisons between factor levels of main effects and significant interaction terms. For all models, we checked normality of residuals by plotting them using the ‘resid’ function.

## 3. Experiment 1: standard judgement bias task paradigm

### 3.1. Experiment 1 methods

In each training trial we presented bees with 12 rewarded (S+; stimulus C) and 12 unrewarded (S−; stimulus F) flowers (figure 1). Bees were trained over a number of trials (2–6) until they reached a performance criterion of 8/10 correct choices on their first 10 choices within a trial. All training trials were video-recorded, but successful completion of training was assessed by live observation. In each trial bees either depleted the array and then returned to the colony, or if 10 min elapsed and the bee had not returned by itself then we returned it to the colony. Individuals that visited fewer than eight flowers on trial one were excluded from the experiment. After training, bees were either given experimental or control treatments before proceeding to the test phase (see *General methods*).

We carried out statistical analysis as described in the *Data analysis* section of the *General methods*. One bee (of 32) was excluded from the analysis since she showed extremely strong preferences to a single location (10/13 (77%) of her visits were to the same location). This resulted in 16 bees in the control treatment and 15 bees in the experimental treatment whose data were included in analyses.

### 3.2. Experiment 1 results and discussion

**Figure 2.**
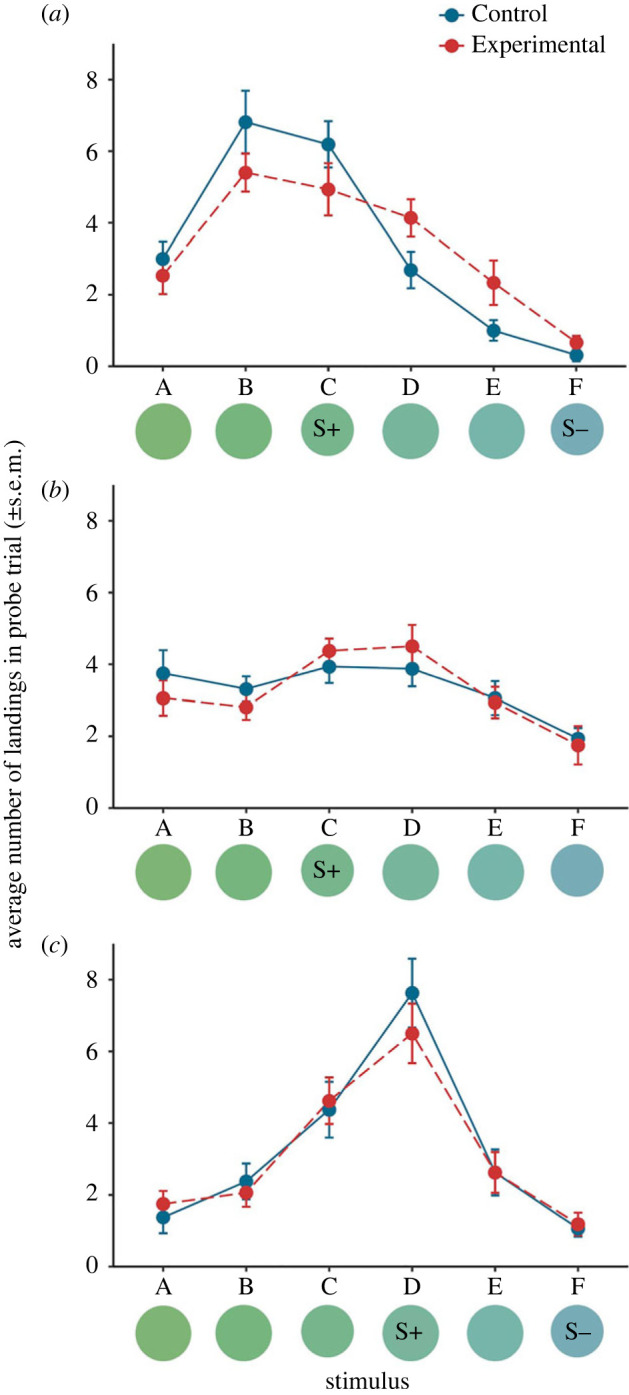
Results of (*a*) Experiment 1 (*b*) Experiment 2 and (*c*) Experiment 3. Bees’ preferences across the six stimuli in the unrewarded probe phase. Bees in the experimental treatment (red, dashed line) experienced a high-quality reward prior to testing, while control bees (blue, solid line) did not. Colour stimuli are shown on the horizontal axis, with the S+ and S− identified for each experiment. In Experiment 1, where bees were trained via differential conditioning, the experimental treatment showed an increased response towards ambiguous stimulus D (*p* = 0.041) and a strong trend towards a decreased response towards stimulus B (*p* = 0.058). The experimental group also appeared to show less peak shift relative to controls. In Experiment 2, where we eliminated the peak shift effect by training bees to the S+ only, both treatments generalized broadly and did not differ from each other. In Experiment 3, where we increased the learned response and eliminated peak shift by training bees on stimuli closer together, treatments also did not differ from each other.

Bees in experimental and control treatments differed in how frequently they visited the six stimuli, as shown by a significant interaction effect and no main effect of Condition (Stimulus × Condition: *F*_5,130_ = 2.794, *p* = 0.0197; Stimulus: *F*_5,130_ = 34.737, *p <* 0.0001; Condition: *F*_1,25_ = 0.00, *p* = 1.000; figure 2*a*). In line with previous findings [14], bumblebees given a high-quality reward after learning an association demonstrated judgement bias, showing a higher response rate to the ambiguous stimulus D relative to controls, while responding similarly to the trained C (CS+) and F (CS−) stimuli (table 1; figure 2*a*). Bees also appeared to show ‘peak shift’; this is typical of S+/S− discrimination training, where the peak of the response curve is not the S+, but rather a novel stimulus biased away from the S− [36], as has previously been described in bees [24,25,37]. In this case, peak shift was relatively weak but still apparent: bees showed a peak response to not only stimulus C, but also stimulus B shifted away from the S− (no difference in response to stimuli B versus C in a *post hoc* comparison: control group: *t*_130_ = 0.064; *p* = 0.950; experimental group: *t* = *t*_130_ = 0.406; *p* = 0.685). However, the strength of this effect was larger for the control than experimental group, with a strong trend towards more bees choosing stimulus B in the control group (table 1; figure 2*a*). Bees were also more likely to visit particular stimuli on specific probe arrays (Stimulus × Probe array: *F*_15,130_ = 2.010; *p* = 0.0190; Probe array: *F*_3,25_ = 0.000; *p* = 1.000; for details see electronic supplementary material, figure S4 and table S2).

**Table 1.** *Post hoc* differences between control and experimental treatments across the six experimental stimuli in Experiment 1. Results are averaged across the four levels of ‘Probe array’.

stimulus	contrast	estimate	s.e.	d.f.	*t*-ratio	*p-*value
A	control versus experimental	0.517	0.757	25	0.683	0.501
B	control versus experimental	1.505	0.757	25	1.988	0.058
C	control versus experimental	1.119	0.757	25	1.478	0.152
D	control versus experimental	−1.630	0.757	25	−2.153	0.041
E	control versus experimental	−1.191	0.757	25	−1.573	0.128
F	control versus experimental	−0.320	0.757	25	−0.422	0.676

**Figure 3.**
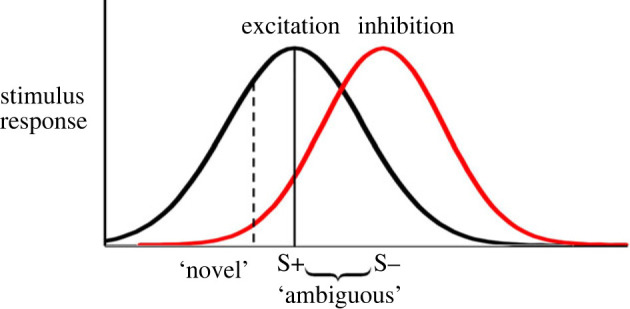
Diagram showing hypothetical excitation (black) and inhibition (red) curves generated by differential training to a S+ and S−. An averaging of these overlapping curves may cause peak shift [38]. On the above diagram, the difference between the excitation and inhibition curves is larger at a value shifted away from the S+ (dashed line) than at the S+ (solid line), and thus there may be a higher response to stimuli at this point.

One explanation for peak shift is that partially or completely overlapping excitation and inhibition gradients average each other out to form the peak-shifted shape of the response curve [38] (figure 3). Since a peak shift response is driven by individuals’ training to the S− [35], our finding that peak shift was lessened by the pre-test reward may indicate that the experimental treatment altered the gradient around the S−, for example by narrowing it. To test the hypothesis that the pre-test sucrose drove changes to individuals’ responses to ambiguous stimuli via effects on the peak shift response, we conducted Experiment 2. Since inhibitory response curves cannot be easily measured directly, we aimed to eliminate the peak shift response in the control group with the prediction that its elimination should also eliminate the difference between the two experimental groups. To do this, we trained bees via absolute conditioning (S+ only), thus removing the inhibitory response curve completely. Bees then underwent the same experimental manipulation and probe trial as Experiment 1.

## 4. Experiment 2: judgement bias task with absolute conditioning

In this experiment, we tested the hypothesis that our results from Experiment 1 were due to a reduction or elimination of the peak shift response. To do this, we trained bees via absolute conditioning; without a S−, no inhibitory curve would be generated and thus no peak shift response. We predicted that if our hypothesis held, the differences between the treatments would be eliminated when peak shift was eliminated.

### 4.1. Experiment 2 methods

Bees exclusively experienced the S+ (C) stimuli during training (figure 1); S− stimuli were removed and replaced with black rubber stoppers. All bees were given three training trials; this number was chosen since it was the most frequent number of trials needed for bees to reach the learning criterion in Experiment 1 and thus was the closest match possible to the number of rewarded experiences with the S+ that bees would have had in that experiment; a similar calculation was used in [24]. Once bees completed training and returned to the colony they were assigned to experimental or control treatments and were given probe trials as in Experiment 1.

We carried out statistical analysis as described in the *Data analysis* section of the *General methods*. Four bees made fewer than 20 visits (control *n* = 2; experimental *n* = 2); these varied from 14 to 19 visits and were included, meaning a final sample size of 16 experimental and 16 control bees included in data analyses.

### 4.2. Experiment 2 results and discussion

Without the S−, bees generalized broadly across stimuli, widening the stimulus response curve (figure 2*b*). In support of our hypothesis, control and experimental groups did not differ from each other (Condition: *F*_1,27_ = 0.0737, *p* = 0.788). However, while bees generally chose the S+ above other stimuli (Stimulus: *F*_5,155_ = 0.7.011, *p* < 0.001), learning was relatively weak, with bees choosing the S+ stimulus above B, E and F stimuli (significant effect or trend with *post hoc* pair-wise comparisons), but not above stimuli A or D (table 2). In this experiment the probe array used did not affect bees’ choice of stimuli (Probe array: *F*_2,27_ = 0.0784, *p* = 0.925).

**Table 2.** *Post hoc* differences between the S+ (C) stimulus and five other stimuli in Experiment 2. Results are averaged across the two levels of the factor ‘Condition’ and four levels of the factor ‘Probe array’.

contrast	estimate	s.e.	d.f.	*t*-ratio	*p*-value
C–A	−0.750	0.463	155	−1.62	0.353
C–B	−1.094	0.463	155	−2.362	0.080
C–D	0.031	0.463	155	0.067	1.000
C–E	−1.156	0.463	155	−2.497	0.058
C–F	−2.313	0.463	155	−4.994	<0.001

While the results of this experiment supported our hypothesis, we only saw weak learning in both treatments. That absolute conditioning only led to weak learning is in line with previous work showing that differential conditioning is necessary for fine-scale colour discrimination [39]. However, other work addressing bumblebee generalization gradients found equivalent learning when bees were trained via absolute conditioning using the average number of training sessions taken by groups that received differential conditioning [24]; differences between that study and our own are probably due to subtle differences in training regime or stimulus discriminability.

To tackle this limitation, in Experiment 3 we aimed to determine if our hypothesis held when bees showed a stronger learned response. To increase learning while manipulating the peak shift response, we decreased the distance between our S+ and S− stimuli. While decreasing the distance between stimuli can increase the peak shift if curves overlap more with each other [40], training animals to stimuli closer together (i.e. requiring finer discriminability) typically reduces stimulus generalization [39,40] and could also reduce or eliminate peak shift if the excitation and inhibition curves narrowed to the point of not overlapping. If our ‘peak shift hypothesis’ as an explanation for judgement bias effects held, we predicted that if peak shift increased, we would increase the difference between our treatments, whereas if peak shift decreased, we would decrease the difference between our treatments.

## 5. Experiment 3: judgement bias task with reduced distance between the trained stimuli

### 5.1. Experiment 3 methods

Bees underwent the same training and testing procedures in Experiment 3 as for Experiment 1, with the exception that the rewarded stimulus (S+) was stimulus D and the unrewarded stimulus (S−) was stimulus F.

We carried out statistical analysis as described in the *Data analysis* section of the *General methods*. Ten bees made fewer than 20 visits (control *n* = 4; experimental *n* = 6); these varied from 12 to 19 visits and were included in the final data analysis.

### 5.2. Experiment 3 results and discussion

When we trained bees to a S+ and S− closer together, it narrowed the stimulus response curve, causing a strong learned response to the S+ and no peak shift (figure 2*c*, table 3). Accordingly, bees visited the S+ (D) stimulus more than all other stimuli (Stimulus: *F*_5,145_ = 29.629, *p <* 0.0001; table 3). In support of our hypothesis, once peak shift was eliminated, there was no difference between experimental and control treatments in the stimuli bees visited (Condition: *F*_1,27_ = 0.117, *p* = 0.735). As in Experiment 1, we found that certain stimuli were preferred on particular probe arrays (Stimulus × Probe array: *F*_10,145_ = 1.910, *p* = 0.0482; Probe array: *F*_2,27_ = 0.084; *p* = 0.920); electronic supplementary material, figure S5 and table S3), probably due to location preferences.

**Table 3.** *Post hoc* differences between the S+ (D) stimulus and five other stimuli in Experiment 3. Results are averaged across the two levels of the factor ‘Condition’ and four levels of the factor ‘Probe array’.

contrast	estimate	s.e.	d.f.	*t-*ratio	*p-*value
D–A	−5.67	0.595	145	−9.532	<0.0001
D–B	−4.99	0.595	145	−8.384	<0.0001
D–C	−2.95	0.595	145	−4.951	<0.0001
D–E	−4.62	0.595	145	−7.756	<0.0001
D–F	−6.06	0.595	145	−10.187	<0.0001

## 6. General discussion

The discovery of emotion-like states in invertebrates raises questions about the evolutionary origin of emotions, as well as having implications for animal welfare. A cognitive framework for the study of judgement bias may help explain discrepancies between previous studies and offer a new perspective on interspecific comparisons. By addressing bees’ generalization curves after receiving a reward, we found that rewarded individuals showed an increased response to an ambiguous stimulus, but a decreased response to a novel stimulus. As such, this group showed a lessened peak shift response across a gradient of stimuli. When we manipulated the training procedure to eliminate peak shift, we no longer found a difference between experimental and control treatments in their response to any stimuli.

Taken together, our experiments support the hypothesis that behaviour observed towards ambiguous stimuli during ‘optimistic’ judgement bias protocols can be explained by changes to the peak shift effect of stimulus response curves, presumably through a change to the inhibitory response curve around the S−. Since we did not measure the S− curve directly we cannot be certain as to how it changed, however, one possibility is that the experimental treatment in Experiment 1 narrowed it; this would explain the reduction of peak shift while response to the S− remained unchanged. This explanation would be consistent with our manipulation in Experiment 3, which we believe also narrowed the inhibitory generalization curve.

One explanation for previously reported judgement bias effects in bees [14] is that a high-quality sucrose reward after training may increase foraging motivation in general, and in doing so increase responsiveness to ambiguous stimuli [15], as well as novel stimuli (as tested in our original hypothesis in Experiment 1). Indeed, high-quality sucrose generally motivates bees’ foraging: experimental addition of sucrose into a colony can trigger foraging, with higher-concentration sucrose causing greater foraging activity [41]. However, our findings do not support this explanation for judgement bias effects, since this would have led to a higher response to the similar novel stimulus B in Experiment 1 and stimuli similar to the S+s in Experiments 2 and 3. Finally, we also found that another potential measure of foraging motivation, the bees’ time to visit the array, did not differ between experimental and control treatments in any of our experiments. Another possibility for an increase in visitation to ambiguous stimuli following a high-quality sucrose reward could be increased sampling. Receiving a high-quality food reward, either in the colony, or in this case, on the outside of the colony, may give individuals information that there is higher-quality food in their environment [42,43]. We would expect this to lead to greater search on ‘novel’ flowers that were not previously associated with a particular reward value, since bees form specific expectations of reward quality based on the associated stimuli [44,45]. However, this would not explain our results either: bees that experienced the higher-quality sucrose reward did not visit novel stimuli A and B more in Experiment 1, nor any novel stimuli more in Experiments 2 and 3.

Judgement bias is typically assessed by offering an animal the two trained stimuli and ambiguous stimuli between these; our method and framework of offering a wider range of stimuli to determine stimulus generalization curves may be a useful means to address why and how different types of experience alter animals’ stimulus perception. Judgement biases are probably a result of several different processes (reviewed in [3], section 4). For example, factors known to affect discrimination training (the distance between the trained S+ and S− stimuli, salience of stimuli etc. [46]) will determine generalization curves post initial training. These curves may then be altered by affective state. A better understanding of how particular experiences change generalization curves (e.g. chronic versus acute stress, the context of the reward or punishment, the timing of the experience relative to training) may shed light on discrepancies in results from judgement bias tasks [6] and provide a framework for generating predictions in future work. Existing knowledge of how affective states affect learning and generalization may be useful in this regard. For example, classic work in experimental psychology shows that pigeons that are hungrier have broader stimulus generalization gradients [17]. If this is a general phenomenon, it could explain why chronically food-restricted sheep *Ovis aries* behaved more ‘optimistically’ on a judgement bias task [47] and why dogs *Canis familiaris* fed prior to testing had seemingly ‘pessimistic’ judgements, in the opposite directions to what was expected based on the animals' presumed emotional states [48]. These examples highlight how, without a mechanistic framework to understand judgement bias, we risk misinterpreting animals’ emotional states. Changes to generalization curves may also explain why in some studies the response to the S− changes in addition to the response to ambiguous stimuli: if a negative experience increases the peak of the inhibitory curve as well as broadening it, the experimental group would respond more to the S− in addition to ambiguous stimuli, as has been found for honeybees stressed via shaking [5] and sheep given chronic stress in an agricultural setting [49]. Indeed, stressed states can both lead to higher responsiveness to negative stimuli and overgeneralization of responses (reviewed in [50,51]). The timing of the affective state manipulation may also affect the learned response; many judgement bias studies manipulate state during learning (reviewed in [6]), rather than post-learning as in the current study, and the state an animal is in when learning can affect how it perceives the relative value of the reward (e.g. [52]) and thus determine learning and generalization curves. Another consideration in predicting how a generalization curve may change in response to an experimental manipulation is the type of stimulus used in differential conditioning. In the current study, we focused on colour, a ‘rearrangement dimension’ [40], where the generalization gradient forms a Gaussian curve. Other stimuli follow an ‘intensity dimension’, where the same receptors are stimulated to a different extent, for example light intensity or tone frequency [40]. In these cases, animals may not form a Gaussian curve when generalizing, but instead always respond more to higher intensities of the stimulus, and as such show a different peak shift response [40,53]. Finally, Spence's model of peak shift [38] that we assume here (figure 3) is only one explanation for how peak shift occurs. Going forward, other models could be considered in terms of how we would expect animals’ affective state to alter stimulus generalization [40,54].

In addition to expanding the range of stimuli used, we also adopted a different protocol to one that is typically used in judgement bias tasks in the probe trial phase of our experiment. Previous research on judgement biases has been conducted using a variety of methods that vary across all phases of the experiment, including discrimination training, experimental manipulation and the probe trial [6], all of which may affect interpretation of differences in results across studies [55]. In regard to the probe trial, previous work has either presented animals with a single stimulus at a time and measured propensity or latency to perform an action (‘go/no-go’ design), or measured ‘active choice’ between two options, as a means of controlling for motivation, since a response is necessary for any choice made [3,8,9,55,56]. Furthermore, in some experiments (as in the original experiment investigating judgement bias in bumblebees [14]), the experimental manipulation is given before each stimulus presentation in the probe trial, whereas in other experiments, multiple stimuli are presented sequentially in the probe phase, without repeating the experimental manipulation between exposures (e.g. [11,57]). In our experiment, bees visited 20 unrewarding stimuli in succession; we designed our experiment this way in line with previous work on generalization gradients [24,25]. However, a clear next step would be to determine if our results hold in other paradigms, for example if stimuli are presented sequentially rather than simultaneously, and in go/no-go designs.

In conclusion, our results imply that good or bad experiences may alter how animals classify a range of stimuli in their worlds, and not only the ‘ambiguous’. Thus, ‘optimism’ and ‘pessimism’ may not simply be a change in animals’ perception of the uncertain, but rather a shift in how many stimuli are perceived. Moving forward, our suggestion that changes to peak shift underlie positive judgement bias provides a framework for advancing the comparative study of emotions. It also serves to reinvigorate investigation of how animals’ experiences can influence psychophysical aspects of learning tasks. Given the central role of judgement bias tasks in topics of animal emotion and welfare, it is essential that we understand the underlying cognitive mechanisms to avoid superficial intra- and interspecific comparisons that may lead to inaccurate equivalence.

## Data Availability

The data are available from the Dryad Digital Repository: https://doi.org/10.5061/dryad.51c59zwd5 [58]. Supplementary material is available online [59].
